# The Association Between Serum Mature and Precursor Brain-Derived Neurotrophic Factor and Neurocognitive Function in People With Human Immunodeficiency Virus: A Longitudinal Study

**DOI:** 10.1093/ofid/ofae463

**Published:** 2024-08-14

**Authors:** Henry U Michael, Antony M Rapulana, Theresa Smit, Njabulo Xulu, Sivapragashini Danaviah, Suvira Ramlall, Frasia Oosthuizen

**Affiliations:** Discipline of Pharmaceutical Sciences, School of Health Science, University of KwaZulu-Natal, Durban, South Africa; Centre for Outcomes Research and Evaluation, Research Institute of McGill University Health Centre, Montreal, Quebec, Canada; Africa Health Research Institute, Durban, South Africa; School of Laboratory Medicine and Medical Science, University of KwaZulu-Natal, Durban, South Africa; UCL Centre for Clinical Microbiology, Division of Infection and Immunity, University College London, London, United Kingdom; Africa Health Research Institute, Durban, South Africa; Africa Health Research Institute, Durban, South Africa; Faculty of Applied Science, Eduvos, Midrand, South Africa; Department of Psychiatry, University of KwaZulu-Natal, Durban, South Africa; Discipline of Pharmaceutical Sciences, School of Health Science, University of KwaZulu-Natal, Durban, South Africa

**Keywords:** brain-derived neurotrophic factor, HIV, neurocognition, proBDNF, sub-Saharan Africa

## Abstract

**Background:**

Despite antiretroviral therapy (ART), human immunodeficiency virus (HIV)–associated neurocognitive impairment persists. We investigated the association between serum levels of mature brain-derived neurotrophic factor (mBDNF), precursor brain-derived neurotrophic factor (proBDNF), and neurocognitive changes over time among adults with HIV in sub-Saharan Africa, seeking to elucidate the interplay between neurotrophic factors and neurocognitive outcomes post-ART.

**Methods:**

Utilizing data from the ACTG 5199 study in Johannesburg and Harare, serum mBDNF and proBDNF levels were measured via enzyme-linked immunosorbent assay. Neurocognitive performance was assessed at baseline and 24, 48, and 96 weeks using neuropsychological tests. The Friedman test and linear mixed-effects models were used to assess changes in mBDNF, proBDNF, and neurocognitive performance over time, accounting for individual variability and adjusting for multiple comparisons.

**Results:**

Among 155 participants, there were significant cognitive improvements (*P* < .001) and a rise in mBDNF levels from baseline to 96 weeks. The proBDNF levels initially remained stable (*P* = .57) but notably increased by 48 weeks (*P* = .04). Higher mBDNF levels were positively associated with enhanced neurocognitive performance at 48 weeks (β = .16, *P* = .01) and 96 weeks (β = .32, *P* < .001). Similarly, higher proBDNF levels were positively associated with neurocognitive performance at 96 weeks (β = .25, *P* < .001).

**Conclusions:**

This study highlights the significant association between serum BDNF levels and neurocognitive improvement post-ART in adults with HIV. However, more research is needed to replicate these findings, establish causal relationships, and explore whether BDNF-enhancing activities can improve neurocognitive outcomes in people with HIV.

Despite reduced mortality from human immunodeficiency virus (HIV)/AIDS in the era of antiretroviral therapy (ART), HIV-associated neurocognitive impairment remains prevalent, affecting 43%–46% of individuals [1–3]. In resource-limited settings, prevalence rates vary widely from 12% to 74% [4], reflecting differences in diagnostic methods, definitions of impairment, and the influence of biopsychosocial factors. Although often asymptomatic or mild [5], such impairments commonly affect verbal fluency, motor skills, processing speed, attention, memory, and executive functions [6].

Brain-derived neurotrophic factor (BDNF), the most abundant growth factor in the central nervous system (CNS), is significantly implicated in HIV-associated neurocognitive impairment [7–9]. Beyond the CNS, BDNF is present in peripheral tissues such as skeletal muscles, adipose tissue, endothelial cells, and platelets [10]. It originates as precursor BDNF (proBDNF) and is subsequently proteolytically cleaved into mature BDNF (mBDNF). This mature form is crucial for neurogenesis, synaptic plasticity, and long-term potentiation essential for learning and memory [11, 12]. Conversely, its precursor, proBDNF, leads to axonal retraction, dendritic degeneration, and long-term depression, embodying the “yin-yang” hypothesis of neuroplasticity [13]. This dual functionality highlights BDNF's complex role in both fostering neural development and contributing to neurocognitive impairments associated with HIV [14, 15]. In this report, the term “BDNF” refers to either the total BDNF or in cases where distinctions between mBDNF and proBDNF are not made clear.

Clinical research indicates that reduced levels of BDNF in cerebrospinal fluid, serum, and plasma are linked to neurocognitive decline in individuals with HIV [7, 16–19]. These studies, however, have limitations. Primarily, their cross-sectional nature offers mechanistic insights but lacks the ability to determine causality, disease progression, or the effects of interventions like ART on BDNF levels and neurocognitive outcomes. Notably, except for 1 study identifying a proBDNF/mature BDNF ratio correlation with neurocognitive impairment in postmortem brain samples of individuals with HIV [7], existing research does not differentiate between mature BDNF and proBDNF. Furthermore, the influence of ART on BDNF levels and its subsequent effects on cognition has not been thoroughly examined in published studies whose participants are either ART-naive or prevalent users. This gap is significant, considering animal studies indicate ART may lower BDNF levels, potentially impacting neurocognitive function [20, 21]. Additionally, increased BDNF is linked to neuropathic pain from nucleoside reverse transcriptase inhibitors in mice, influencing motor skills [22]. These findings highlight the complex interplay between ART, BDNF, and neurocognitive function, underscoring the need for more nuanced research.

In this study, we aimed to estimate the association between serum mBDNF and proBDNF with neurocognitive performance over 96 weeks post–ART initiation in a cohort of people with HIV in sub-Saharan Africa. Our research questions were: (1) How do serum mBDNF and proBDNF levels change post–ART initiation in people with HIV? (2) How do changes in serum levels of mBDNF and proBDNF influence neurocognitive function over the course of ART in people with HIV? We hypothesized that an increase in mBDNF and a decrease in proBDNF levels post-ART would be associated with improved neurocognitive performance. This secondary analysis utilized data from the International Neurological Study AIDS Clinical Trials Group (ACTG) 5199, comparing neurological outcomes across 3 antiretroviral regimens in resource-limited settings [23, 24].

## MATERIALS AND METHODS

### Participants

The sample for our study was selected from enrolled participants at the Johannesburg and Harare sites of the ACTG A5199 study, conducted from February 2006 to May 2010. This randomized controlled trial evaluated neurocognitive and neurological effects of 3 ART regimens [23–25]. As this was a secondary analysis, more details of the study design have been provided elsewhere [23–25]. Participants were adults 18 years or older with HIV, antiretroviral-naive with CD4^+^ counts <300 cells/μL, having a Karnofsky score of ≥70, and having no serious illness, psychiatric condition, or acute substance dependence/abuse. All participants commenced ART and were followed every 24 weeks for up to 192 weeks with neurocognitive assessments, standardized interviews, and laboratory tests. This analysis focused on participants with baseline and at least 1 follow-up neurocognitive test and serum sample at 24, 48, or 96 weeks. All procedures were approved by each participating site and the university institutional review board.

### Measurement of BDNF

Serum levels of mBDNF and proBDNF were analyzed using a specific enzyme-linked immunoassay kit following standardized manufacturer's protocols (Biosensis Pty Ltd, Thebarton, Australia). The detection ranges for the assays were 7.8 to 500 pg/mL for mBDNF and 15.6 to 1000 pg/mL for proBDNF. Stored at −70°C, samples were assayed in duplicate on the same analytical plate to ensure uniformity and minimize interassay variability. The intra-assay variances for mBDNF and proBDNF were low, at 2.7% and 2.2%, respectively, ensuring high reliability of measurements. Furthermore, the laboratory personnel conducting the assays were blinded to participants' clinical and neurocognitive data to prevent bias.

### Neuropsychological Assessment

Four neuropsychological tests were administered by trained psychologists [25]: timed gait for motor speed, semantic verbal fluency for language and executive function, grooved pegboard for fine motor skills and coordination (both hands), and finger tapping for motor speed and coordination (both hands). These tests were selected based on previous experience and suitability for a culturally and linguistically diverse cohort in a global, resource-varied setting [23]. Baseline scores and follow-ups at 24, 48, and 96 weeks were standardized into *z*-scores based on demographic adjustments from the International Neurocognitive Normative Study ACTG A5271, representing at-risk demographics [26]. These *z*-scores were averaged to create a composite score, with higher scores reflecting better neurocognitive function.

### Covariates

Study covariates included baseline age (years), education (years), biological sex, CD4 count (cells/μL), and log-transformed HIV RNA levels.

### Statistical Analysis

Statistical analyses were conducted using R software version 4.3.1 (R Core Team, 2023, Vienna, Austria). Descriptive statistics summarized demographics, clinical data, neuropsychological test scores, and log-transformed mBDNF and proBDNF levels (pg/mL) at baseline, 24, 48, and 96 weeks, presented as median with interquartile range (IQR). The normality of continuous variables was assessed using the Kolmogorov-Smirnov test. The Friedman test evaluated changes in CD4 count, CD8 count, log_10_-transformed plasma HIV RNA, BDNF levels, and neurocognitive performance over time, with Wilcoxon signed-rank tests for post hoc analysis, adjusted using the Holm-Bonferroni method for multiple comparisons.

Linear mixed-effects models, incorporating fixed effects and random effects (intercept and slope), were used to explore the relationship between serum mBDNF and proBDNF levels and neurocognitive function over time. Repeated measures of neurocognitive test scores, including both composite neurocognitive score (NPZ-6) and specific tests, served as outcomes, with log-transformed mBDNF and proBDNF levels as the main predictors. The interaction term between time and mBDNF and proBDNF levels assessed the influence of BDNF on neurocognitive trajectory. A positive interaction coefficient indicated that higher mBDNF levels were associated with neurocognitive improvement (similar for proBDNF). A random intercept was specified for each participant to address individual-specific variability. Models were adjusted for age, education, sex, CD4/CD8 ratio, and log-transformed plasma HIV RNA to control for potential confounders.

Model assumptions, including normality, homoscedasticity, independence, and the influence of outliers, were checked using plots of residuals against fitted values (to assess homoscedasticity and nonlinearity) and quantile-quantile (Q-Q) plots (to assess normality of residuals). Robust linear mixed-effects modeling was performed to address common violations of classical linear mixed-effects modeling assumptions in longitudinal studies with repeated neuropsychological measures [27–29]. This method attenuated the impact of observations exhibiting significant residuals or random effects, thereby mitigating their influence on model estimates. *P* values and 2-sided 95% confidence intervals of the parameter estimates for the main predictors, interaction terms, fixed effects, and random effects in the linear mixed-effects models were computed using bootstrapping techniques to assess the precision and reliability of the associations between BDNF levels and neurocognitive function over time [30]. In cases where disparities in effect magnitude, direction, or statistical significance arose, precedence was given to the outcomes of the robust linear mixed-effects model [29].

All analyses were conducted using the “lme4” [31] and “robustlmm” [28] packages in R, with statistical significance set at *P* < .05, adjusted for multiple comparisons using the Holm-Bonferroni method.

### Sensitivity Analysis

In our primary analysis, time post-ART was treated as a categorical variable to identify discrete changes in overall neurocognitive performance at specified intervals, revealing potential nonlinear effects of ART. For the sensitivity analysis, we recoded time post-ART as a numeric variable, enabling us to evaluate the general linear progression of neurocognitive changes over the ART duration. This alternative method enhanced our analysis by juxtaposing detailed, interval-specific observations with overarching linear trends. Rigorous statistical evaluations were applied to both models, ensuring their validity, and a comparative review of findings from both approaches underscored the reliability of our conclusions.

## RESULTS

One hundred fifty-eight participants provided baseline blood samples and neuropsychological data. After excluding 3 participants with no follow-up data, 155 participants were included in the final longitudinal analysis. Of these, 136 had complete data across the 4 timepoints, and 19 had data for at least 2 time points.

### Descriptive Statistics

Table 1 details the demographic and clinical characteristics of participants. Nearly half (49.03%) were from the Johannesburg site. Participants, on average, were 36.11 years old (standard deviation, 7.54 years), with males comprising 34.19% of the sample. Median education was 11 years (IQR, 9–12 years), and the median CD4 count at baseline was 191.50 cells/μL (IQR, 130.25–228.50 cells/μL). Table 2 details the changes in clinical characteristics of participants. Log-transformed plasma HIV RNA decreased significantly from baseline (*P* < .001), stabilizing thereafter. CD4 cell counts increased significantly over time (*P* < .001), with each subsequent measure improving. CD8 cell counts decreased significantly by 96 weeks compared to baseline (*P* = .002), with no significant change between 48 and 96 weeks (*P* = .25). CD4/CD8 ratio increased significantly (*P* < .001), indicating immune system recovery with ART.

**Table 1.** Baseline Characteristics of Participants

Characteristics	N = 155
Personal characteristics	
Age, y, mean (SD)	36.11 (7.54)
Sex, male, No. (%)	53 (34.19)
Education, y, median (IQR)	11 (9–12)
Johannesburg site, No. (%)	76 (49.03)
HIV-related variables, median (IQR)	
Log_10_ plasma HIV RNA, copies/mL	5.02 (4.51–5.49)
CD4, cells/μL	191.50 (130.25–228.50)
CD8, cells/μL	817.50 (582.50–1191.25)
CD4/CD8 ratio, cells/μL	0.20 (0.13–0.29)
ART regimen, No. (%)	
EFV/3TC/ZDV	55 (35.48)
FTC/ATV/ddI-EC	58 (37.42)
EFV/FTC/TDF	42 (27.1)
Serum BDNF, median (IQR)	
Log_10_ BDNF, pg/mL	1.95 (1.86–2.03)
Log_10_ proBDNF, pg/mL	1.61 (1.15–2.16)
Neuropsychological *z*-scores, median (IQR)	
Composite (NPZ-6)	0.19 (−0.4 to 0.7)
Grooved pegboard (dominant hand)	−0.20 (−0.90 to 0.34)
Grooved pegboard (nondominant hand)	−0.14 (−0.89 to 0.29)
Finger tapping (dominant hand)	0.70 (−0.18 to 1.78)
Finger tapping (nondominant hand)	0.57 (−0.49 to 1.77)
Timed gait	−0.61 (−1.53 to 0.32)
Semantic verbal fluency	0.27 (−0.71 to 1.94)

Abbreviations: 3TC, lamivudine; ATV, atazanavir; BDNF, brain-derived neurotrophic factor; ddI-EC, didanosine enteric-coated; EFV, efavirenz; FTC, emtricitabine; HIV, human immunodeficiency virus; IQR, interquartile range; SD, standard deviation; TDF, tenofovir disoproxil fumarate; ZDV, zidovudine.

**Table 2.** Longitudinal Changes in Clinical Characteristics of Participants

Characteristics	Baseline (0 wk) (N = 155)	24 wk (N = 155)	48 wk (N = 146)	96 wk (N = 138)	*P* Value^a^	Post Hoc Comparisons^b^
Log plasma HIV RNA, copies/mL	5.02 (4.51–5.49)	2.60 (2.60–2.60)	2.60 (2.60–2.60)	2.60 (2.60–2.60)	<.001***	24 wk vs 0 wk: <.001*** 48 wk vs 0 wk: <.001*** 96 wk vs 0 wk: <.001*** 48 wk vs 24 wk: .66 96 wk vs 24 wk: 1 96 wk vs 48 wk: 1
CD4, cells/μL	191.50 (130.25–228.50)	300.00 (231.50–398.50)	336.00 (245.75–433.75)	395.00 (309.25–499.75)	<.001***	All comparisons: <.001***
CD8, cells/μL	817.50 (582.50–1191.25)	797.00 (598.50–1108.00)	780.50 (536.50–995.25)	749.50 (554.25–954.75)	<.001***	24 wk vs 0 wk: .25 48 wk vs 0 wk: .01* 96 wk vs 0 wk: .002** 48 wk vs 24 wk: .02* 96 wk vs 24 wk: .002** 96 wk vs 48 wk: .25
CD4/CD8 ratio	0.20 (0.13–0.29)	0.37 (0.28–0.49)	0.44 (0.31–0.60)	0.55 (0.40–0.71)	<.001***	All comparisons: <.001***

Data are presented as median (interquartile range).

Abbreviation: HIV, human immunodeficiency virus.

^a^Friedman test was conducted to assess changes over time.

^b^Post hoc comparisons using Wilcoxon signed-rank tests were adjusted for multiple comparisons using the Holm-Bonferroni method.

^*^Indicates significance at *P* < .05.

^**^Indicates significance at *P* < .01.

^***^Indicates significance at *P* < .001.

### Changes in Serum mBDNF and proBDNF

Table 3 outlines the changes in serum mBDNF and proBDNF over the analysis period. Serum mBDNF exhibited a notable increase from baseline at 24 weeks post–ART initiation (*P* < .001), maintaining elevated levels at 48 weeks (*P* < .001) and 96 weeks (*P* < .001). Importantly, the rate of increase between the 24-week and 48-week time points (*P* = .11), as well as between the 24-week and 96-week assessments (*P* = .21), remained consistent, indicating a steady upward trend throughout the study.

**Table 3.** Changes in Serum Mature Brain-Derived Neurotrophic Factor (BDNF), proBDNF, and Cognitive Test Scores

Measures	Baseline (0 wk) (N = 155)	24 wk (N = 155)	48 wk (N = 146)	96 wk (N = 138)	*P* Value^a^	Post Hoc Comparisons^b^
BDNF, median (IQR)						
Serum log_10_ mBDNF, pg/mL	1.95 (1.86–2.03)	2.06 (1.97–2.20)	2.04 (1.94–2.16)	2.06 (1.98–2.17)	<.001***	24 wk vs 0 wk: <.001*** 48 wk vs 0 wk: <.001*** 96 wk vs 0 wk: <.001*** 48 wk vs 24 wk: .11 96 wk vs 24 wk: .21 96 wk vs 48 wk: .46
Serum log_10_ proBDNF, pg/mL	1.61 (1.15–2.16)	1.40 (0.93–2.08)	1.66 (1.11–2.07)	1.99 (1.55–2.39)	<.001***	24 wk vs 0 wk: .57 48 wk vs 0 wk: .04* 96 wk vs 0 wk: .75 48 wk vs 24 wk: .51 96 wk vs 24 wk: .57 96 wk vs 48 wk: .14
Cognitive function, median (IQR)						
Composite (NPZ-6)	0.19 (−0.40 to 0.70)	0.33 (−0.26 to 0.72)	0.42 (−0.14 to 0.96)	0.75 (0.14–1.29)	<.001***	24 wk vs 0 wk: .008** 48 wk vs 0 wk: <.001*** 96 wk vs 0 wk: <.001*** 48 wk vs 24 wk: .008** 96 wk vs 24 wk: <.001*** 96 wk vs 48 wk: <.001***
Grooved pegboard (dominant hand)	−0.20 (−0.90 to 0.34)	0.10 (−0.57 to 0.54)	0.21 (−0.26 to 0.61)	0.40 (−0.09 to 0.80)	<.001***	24 wk vs 0 wk: <.001*** 48 wk vs 0 wk: <.001*** 96 wk vs 0 wk: <.001*** 48 wk vs 24 wk: .07 96 wk vs 24 wk: <.001*** 96 wk vs 48 wk: .006**
Grooved pegboard (nondominant hand)	−0.14 (−0.89 to 0.29)	0.08 (−0.50 to 0.59)	0.22 (−0.49 to 0.64)	0.26 (−0.23 to 0.70)	<.001***	24 wk vs 0 wk: <.001*** 48 wk vs 0 wk: <.001*** 96 wk vs 0 wk: <.001*** 48 wk vs 24 wk: .43 96 wk vs 24 wk: .02* 96 wk vs 48 wk: .23
Finger tapping (dominant hand)	0.71 (−0.18 to 1.78)	1.32 (0.47–2.29)	1.20 (0.09–2.28)	1.21 (0.13–2.08)	<.001***	24 wk vs 0 wk: <.001*** 48 wk vs 0 wk: .01* 96 wk vs 0 wk: .004** 48 wk vs 24 wk: .48 96 wk vs 24 wk: .48 96 wk vs 48 wk: .76
Finger tapping (nondominant hand)	0.57 (−0.49 to 1.77)	1.04 (0.19–2.05)	0.82 (−0.01 to 2.02)	0.74 (−0.13 to 1.87)	<.001***	24 wk vs 0 wk: <.001*** 48 wk vs 0 wk: .04* 96 wk vs 0 wk: .09 48 wk vs 24 wk: .37 96 wk vs 24 wk: .27 96 wk vs 48 wk: .45
Timed gait	−0.61 (−1.53 to 0.32)	−0.09 (−1.10 to 0.58)	−0.09 (−1.14 to 0.58)	−0.09 (−1.01 to 1.22)	<.001***	24 wk vs 0 wk: <.001*** 48 wk vs 0 wk: .06 96 wk vs 0 wk: <.001 48 wk vs 24 wk: .03* 96 wk vs 24 wk: .04* 96 wk vs 48 wk: <.001
Semantic verbal fluency	0.27 (−0.71 to 1.94)	−0.49 (−1.32 to 0.18)	0.45 (−0.65 to 1.73)	0.53 (−0.62 to 2.29)	<.001***	24 wk vs 0 wk: <.001*** 48 wk vs 0 wk: .5 96 wk vs 0 wk: .01* 48 wk vs 24 wk: <.001 96 wk vs 24 wk: <.001 96 wk vs 48 wk: .1

Abbreviations: BDNF, brain-derived neurotrophic factor; IQR, interquartile range; mBDNF, mature brain-derived neurotrophic factor; proBDNF, precursor brain-derived neurotrophic factor.

^a^Friedman test was conducted to assess changes over time.

^b^Post hoc comparisons using Wilcoxon signed-rank tests and adjusted for multiple comparisons using the Holm-Bonferroni method.

^*^Indicates significance at *P* < .05.

^**^Indicates significance at *P* < .01.

^***^Indicates significance at *P* < .001.

Conversely, serum log_10_ proBDNF did not experience a statistically significant decrease at 24 weeks compared to baseline (*P* = .57). Although there was a marginal decrease, it did not reach significance. The proBDNF levels exhibited a partial recovery at 48 weeks (*P* = .04) and a subsequent increase at 96 weeks (*P* = .57). No significant differences were observed between the 24-week and 48-week time points (*P* = .75) or between the 24-week and 96-week assessments (*P* = .51), suggesting a stabilization or reversal of the initial decline observed.

### Changes in Neurocognitive Function

Table 3 illustrates the changes in neurocognitive function over the course of the study. Statistically significant improvements were observed across all neurocognitive domains. The composite score, as measured by NPZ-6, showed a significant increase from baseline to 96 weeks post–ART initiation (*P* < .001), indicating consistent enhancement. Individual tests, such as grooved pegboard and finger tapping tasks, and timed gait also exhibited significant improvements from baseline, maintaining gains throughout the study (*P* < .001). Although semantic verbal fluency initially declined, it subsequently improved significantly (*P* < .001), contributing to the overall positive trend in neurocognitive function observed throughout the study.

### Serum mBDNF/proBDNF Levels and Neurocognitive Performance Post–ART Initiation

Table 4 summarizes the relationship between serum mBDNF/proBDNF levels and neurocognitive function post–ART initiation. Two robust linear mixed-effects models were analyzed to investigate serum BDNF's role in neurocognitive change over time. Model 1 focused on log_10_-transformed BDNF (mBDNF) levels, while model 2 examined log_10_-transformed proBDNF levels. Classical linear mixed-model results are provided in Supplementary Material 1.

**Table 4.** Serum Brain-Derived Neurotrophic Factor Association With Composite Cognitive Score (NPZ-6)

Outcome—Composite Cognitive Score (NPZ-6)				
Variable	β Estimate	SE	t Value	Bootstrapped *P* Value
Model 1—mBDNF				
Log_10_ mBDNF	0.06	0.16	0.35	.73
Log_10_ mBDNF: Week 24	0.1	0.06	1.59	.11
Log_10_ mBDNF: Week 48	0.16	0.06	2.58	.01*
Log_10_ mBDNF: Week 96	0.32	0.06	4.91	<.001***
Age at baseline	0.02	0.007	2.71	.01*
Sex (male)	−0.27	0.11	−2.33	.02*
Years of education	0.05	0.02	2.27	.02*
CD4/CD8 ratio	0.26	0.12	2.21	.03*
Log_10_ HIV RNA	0.05	0.05	1.08	.28
Model 2—proBDNF				
Log_10_ proBDNF	−0.09	0.05	−1.67	.09
Log_10_ proBDNF: Week 24	0.01	0.05	0.26	.8
Log_10_ proBDNF: Week 48	0.07	0.06	1.3	.19
Log_10_ proBDNF: Week 96	0.25	0.06	4.41	<.001***
Age at baseline	0.02	0.01	2.29	.02*
Sex (male)	−0.25	0.12	−2.1	.04*
Years of education	0.05	0.02	2.27	.02*
CD4/CD8 ratio	0.3	0.12	2.55	.01*
Log_10_ HIV RNA	0.0009	0.04	0.03	.98

Each column is estimated from a robust mixed-effects model examining the association of serum BDNF expression with the composite cognitive score (NPZ-6).

Abbreviations: BDNF, brain-derived neurotrophic factor; HIV, human immunodeficiency virus; mBDNF, mature brain-derived neurotrophic factor; proBDNF, precursor brain-derived neurotrophic factor; SE, standard error.

^*^Indicates significance at *P* < .05.

^**^Indicates significance at *P* < .01.

^***^Indicates significance at *P* < .001.

Our analysis from model 1 reveals that initial mBDNF levels did not significantly predict neurocognitive performance (β = .06, *P* = .73). However, as time post–ART initiation progressed, a significant relationship developed. Specifically, at week 48, higher mBDNF levels corresponded with improved neurocognitive scores (β = .16, *P* = .01), with an even stronger association observed by week 96 (β = .32, *P* < .001). Supporting these results, the sensitivity analysis—treating time on ART as a continuous variable—further substantiates the growing positive influence of mBDNF on neurocognitive performance over the treatment period (β = .0031, *P* < .001), as detailed in Supplementary Material 2.

In model 2, while baseline proBDNF levels were not significantly associated with neurocognitive outcomes (β = −.09, *P* = .09), proBDNF levels at week 96 correlated positively with neurocognitive improvement (β = .25, *P* < .001). Sensitivity analysis (see Supplementary Material 2), which considers time on ART as a continuous variable, indicated an initial negative effect of proBDNF on cognition (β = −.12, *P* = .008), followed by an increasing positive impact as ART continues (β = .003, *P* < .001).

Across both models, age at baseline and years of education were positively associated with neurocognitive scores (β = .02 and β = 0.05, respectively, with *P* ≤ .02). Male sex was associated with lower neurocognitive scores (β = −.27 and β = −.25 for models 1 and 2, respectively, with *P* ≤ .04). The CD4/CD8 ratio emerged as a consistent positive predictor (β = .26 for model 1, *P* = .03; β = .3 for model 2, *P* = .01), whereas log_10_ HIV RNA levels did not significantly predict neurocognitive changes.

### Serum mBDNF Association With Neurocognitive Test Scores

Table 5 summarizes the positive association between serum mBDNF and individual neurocognitive test performance. Baseline serum mBDNF levels did not show a significant relationship with the tested neurocognitive functions. However, at week 96, an association was noted with timed gait performance (β = .34, *P* = .02) and grooved pegboard performance for both hands, particularly strong in the dominant hand (β = .28, *P* < .001).

**Table 5.** Serum Brain-Derived Neurotrophic Factor Association With Individual Cognitive Test Scores

Cognitive Tests	β Estimate (SE), Holm-Bonferroni Adjusted *P* Value			
	Log_10_ mBDNF	Log_10_ mBDNF × wk 24	Log_10_ mBDNF × wk 48	Log_10_ mBDNF × wk 96
mBDNF				
Timed gait	0.26 (0.30), 1	0.19 (0.11), .11	0.08 (0.11), .95	0.34 (0.12), .02*
Grooved pegboard (dominant hand)	0.15 (0.18), 1	0.12 (0.06), .11	0.19 (0.07), .02*	0.28 (0.07), <.001***
Grooved pegboard (nondominant hand)	0.33 (0.17), .03*	0.13 (0.06), .09	0.18 (0.06), .02*	0.23 (0.06), .002**
Semantic verbal fluency	−0.04 (0.48), 1	−0.57 (0.18), .01*	−0.08 (0.18), .95	0.20 (0.19), .74
Finger tapping (dominant hand)	0.005 (0.27), 1	0.25 (0.10), .06	0.16 (0.10), .30	0.12 (0.11), .74
Finger tapping (nondominant hand)	0.31 (0.28), 1	0.24 (0.10), .06	0.18 (0.10), .30	0.11 (0.11), .74

proBDNF	Log_10_ proBDNF	Log_10_ proBDNF × wk 24	Log_10_ proBDNF × wk 48	Log_10_ proBDNF × wk 96
Timed gait	−0.16 (0.10), .49	0.19 (0.10), .21	0.03 (0.11), 1	0.31 (0.10), .01*
Grooved pegboard (dominant hand)	0.07 (0.05), .68	0.10 (0.06), .21	0.19 (0.06), .005**	0.24 (0.06), <.001***
Grooved pegboard (nondominant hand)	−0.01 (0.05), .77	0.19 (0.05), .002**	0.21 (0.06), .001**	0.28 (0.06), <.001***
Semantic verbal fluency	0.19 (0.15), .68	−0.92 (0.17), <.001***	−0.32 (0.17), .17	0.01 (0.17), 1
Finger tapping (dominant hand)	−0.12 (0.09), .68	0.15 (0.09), .21	0.01 (0.09), 1	0.01 (0.09), 1
Finger tapping (nondominant hand)	−0.20 (0.09), .14	0.33 (0.10), .003**	0.21 (0.10), .11	0.19 (0.10), .16

All models were adjusted for age, sex, CD4/CD8 ratio, and plasma viral load.

Abbreviations: BDNF, brain-derived neurotrophic factor; mBDNF, mature brain-derived neurotrophic factor; proBDNF, precursor brain-derived neurotrophic factor; SE, standard error.

^*^Indicates significance at *P* < .05.

^**^Indicates significance at *P* < .01.

^***^Indicates significance at *P* < .001.

### Serum proBDNF Association With Neurocognitive Test Scores

Table 5 summarizes the association between serum proBDNF with individual neurocognitive test performance. Baseline proBDNF levels did not predict neurocognitive test performance. Yet, significant improvements were observed in the nondominant hand’s grooved pegboard performance at 24 weeks (β = .19, *P* = .002), with this trend continuing significantly at 48 weeks (β = .21, *P* = .001) and 96 weeks (β = .28, *P* < .001). A decline in semantic verbal fluency was linked to proBDNF at 24 weeks (β = −.92, *P* < .001), but this association did not persist. No significant changes were associated with the finger tapping test at any point.

## DISCUSSION

To our knowledge, this is the first longitudinal study to explore the relationship between neurocognitive improvement and serum BDNF in people with HIV after initiating ART. We found results that diverged from our initial theories and hypotheses about their roles. Both mBDNF and proBDNF were associated with improved neurocognitive performance, with this positive effect becoming significantly evident after 96 weeks of treatment. The proBDNF levels were initially associated with neurocognitive decline but subsequently transitioned to improved neurocognitive performance, aligning with the steady positive association of mBDNF with neurocognitive performance. This indicates a dynamic relationship between these neurotrophic factors and neurocognitive functions over the course of ART. The study not only documents a consistent increase in mBDNF levels, indicating ongoing neuroprotective effects, but also follows a pattern of change in proBDNF levels, with an initial decrease followed by improvement. This suggests their combined roles in neurocognitive changes over time. Furthermore, broad improvements across neurocognitive tests underscore the lasting positive effects of sustained ART on neurocognitive health. These findings enrich our understanding of the complex processes that underpin neurocognitive enhancement within the context of HIV management, adding nuance to the existing body of knowledge.

The significant elevation in neuropsychological test scores from week 48, notably associated with mBDNF levels, corroborates the well-documented role of mBDNF in fostering neural repair and synaptic plasticity [7–9, 32–34]. The initial absence of a predictive link between mBDNF levels and neurocognitive improvements may suggest a latency in the biological underpinnings of mBDNF's efficacy in neurocognitive enhancement, becoming evident only as the effects of ART consolidate over time. Such observations echo findings from research on simian immunodeficiency virus–infected macaques, wherein ART failed to ameliorate BDNF signaling deficits in the frontal cortex and basal ganglia nearly 9.5 months into treatment [35]. Despite our study recording a significant increase in serum mBDNF at 24 weeks following ART initiation, the early neurocognitive improvements observed could be attributed to practice effect, diminished inflammation, and partial immune restoration, as indicated by an improved CD4/CD8 ratio [25, 36–38].

Moreover, the protracted influence of mBDNF on neurocognitive enhancement might also relate to the significantly limited scope of neurocognitive domains assessed by our neuropsychological test battery, which primarily measured motor function and verbal fluency. Interestingly, a study involving individuals with nonpenetrating traumatic brain injury highlighted that serum BDNF levels were linked to memory impairments and neurocognitive functional limitations only 6 months postinjury, suggesting that certain neurocognitive functions might exhibit quicker responses to mBDNF level changes [39]. In our study, semantic verbal fluency, closely tied to executive function [40–42], showed the most rapid response to mBDNF changes at 24 weeks, albeit in a negative direction. This finding prompts further investigation into whether learning, memory, and executive functions are particularly receptive to mBDNF-mediated neurocognitive recovery, necessitating more research to unravel the specific neurocognitive functions that might show accelerated improvement with increased mBDNF levels, thereby deepening our comprehension of neurotrophic factors' roles in neurocognitive recovery amid HIV treatment.

Traditionally, mBDNF is associated with neuroprotective and neurotrophic functions, expected to bolster neurocognitive performance, while proBDNF, linked to neurodegenerative processes, would presumably exert detrimental effects [7]. However, at 96 weeks post-ART, both proteins were found to enhance neurocognitive functions, diverging from this expected dichotomy. An initial association of proBDNF with neurocognitive decline at baseline transitioned to a positive influence, paralleling the beneficial trajectory observed with mBDNF, suggesting a multifaceted interaction within neurotrophic signaling pathways.

The scarcity of longitudinal studies examining proBDNF's impact on neurocognitive performance in the context of HIV complicates direct comparisons. Nonetheless, the broader literature presents mixed findings: Some studies report no link between proBDNF and future neurocognitive outcomes in older adults [43], while others associate increased proBDNF levels with either decline in gait speed [44] or neurocognitive improvements [45–47]. These disparate results hint at a more complex reality diverging from the simplistic “yin-yang” neurotrophin hypothesis.

Several factors may explain these observations. First, an increase in mBDNF and proBDNF levels may serve as a compensatory mechanism to support neurocognitive functions. The production of mBDNF from the cleavage of proBDNF suggests that higher serum levels could indicate increased genetic transcription, providing a larger precursor pool for mBDNF [45, 48].

Second, the influence of mBDNF and proBDNF might be contingent on the phase of neuronal activity, varying with neuron type, cellular health, and the presence of other signaling molecules, pointing to their role being dynamic and context-dependent [49, 50]. Third, methodological considerations also play a role. Serum levels of proBDNF might not accurately reflect CNS activity, given their different cellular origins compared to mBDNF, which is predominantly released during platelet activation. In contrast, circulating proBDNF likely originates from various cell types, not platelets [49, 50]. Studies utilizing extracellular vesicles (EV) enriched for neuronal origin suggest that neuronal-source proBDNF may more accurately represent its levels and effects, as demonstrated by research linking higher levels of neuronal-origin EV-derived proBDNF with declines in gait speed among older adults [44]. Collectively, these findings elucidate the intricate and dynamic contributions of mBDNF and proBDNF to neurocognitive performance, indicating that their effects on neurocognitive health in individuals with HIV on ART may surpass the boundaries set by traditional neurotrophin hypotheses.

Our study presents several strengths, including the longitudinal design, separate assays for proBDNF and mBDNF, robust mixed-effects modeling, and comprehensive sensitivity analysis. The primary and sensitivity analysis, treating time on ART as a categorical and continuous variable, respectively, corroborated each other and provided a nuanced understanding of the temporal dynamics at play. While our study provides valuable insights, it is not without limitations. The sample size constraints prevented us from modeling the effects of specific ART regimens on BDNF levels and cognitive function. The generalizability of our findings may be limited by our cohort's unique demographic and clinical traits, characterized by significant immunosuppression and recruitment from a clinical trial, as well as the specificities of the ART regimens administered. Also, the lack of a comparison group limits our inference as to what long-term results are attributable to mBDNF or BDNF alone, hence our inability to elucidate the specificity of BDNF as a biomarker of HIV-related neurocognitive impairment. Another significant area for improvement is the narrow scope of the neuropsychological test battery, which primarily focuses on motor function and verbal fluency, thereby restricting our ability to draw conclusions beyond these domains.

## CONCLUSIONS

Our study suggests that serum mBDNF and proBDNF levels could be significant indicators of neurocognitive trajectory in the context of ART. Despite the limitations, such as the specific cohort demographics and the narrow scope of neuropsychological tests, our findings contribute valuable insights into the complex role of BDNF in neurocognitive health. Future studies are needed to elucidate these relationships further and explore broader neurocognitive domains.

## Supplementary Data

Supplementary materials are available at *Open Forum Infectious Diseases* online. Consisting of data provided by the authors to benefit the reader, the posted materials are not copyedited and are the sole responsibility of the authors, so questions or comments should be addressed to the corresponding author.

## Supplementary Material

ofae463_Supplementary_Data

## Notes

***Author contributions.*** All authors contributed to the study's conception and design. H. U. M., A. M. R., and N. X. performed material preparation, data collection, and analysis. T. S., S. R., S. D., and F. O. supervised all aspects of the project. The first draft of the manuscript was written by H. U. M., and all authors commented on previous versions. All authors read and approved the final manuscript.

***Acknowledgments.*** The authors thank the study investigators and participants of the ACTG A5175, A5199, and A5271 studies for providing the samples and data for this work. We also thank the ACTG Repository for providing the study specimens (www.specimenrepository.org). The authors also thank the research support staff at the African Health Research Institute, Durban, South Africa, for sample management and testing.

***Ethics approval.*** This study was performed in line with the principles of the Declaration of Helsinki. Approval was granted by the Ethics Committee of University of KwaZulu-Natal (18 August 2022/Number BREC/00003101/2021), University of the Witwatersrand (28 July 2021/04081), and Medical Research Council of Zimbabwe (10 September 2021/MRCZ/E/299).

***Patient consent.*** Informed consent was obtained from all individual participants included in the study.

***Data availability.*** The authors confirm that all data are fully available upon request from sdac.data@sdac.harvard.edu with the written agreement of Advancing Clinical Therapeutics Globally for HIV/AIDS and Other Infections (ACTG). Study specimens were obtained from the ACTG Repository (www.specimenrepository.org).

***Financial support.*** Research reported in this publication was supported by the National Institute of Allergy and Infectious Diseases of the National Institutes of Health (award numbers UM1 AI068634, UM1 AI068636, and UM1 AI106701). H. U. M. received research support from the College of Health Sciences, University of KwaZulu-Natal, Durban, South Africa.
