# Supplementary material for: The Association Between Serum Mature and Precursor Brain-Derived Neurotrophic Factor and Neurocognitive Function in People With Human Immunodeficiency Virus: A Longitudinal Study
Source: Open Forum Infect Dis. 2024 Aug 14;11(8):ofae463. doi: 10.1093/ofid/ofae463 (PMC11347942; doi:10.1093/ofid/ofae463)
Supplement: ofae463_Supplementary_Data [file ofae463_supplementary_data.docx]

**SUPPLEMENTARY MATERIAL**

**S1 Serum mBDNF/ProBDNF Levels and Cognitive Performance Post-ART initiation (Classical Linear Mixed-Model)**

**Mature BDNF**

| Estimate Std. Error df t value Pr(>\|t\|) | | | | | |
| --- | --- | --- | --- | --- | --- |
| Fixed effects: |  |  |  |  |  |
| Estimate Std. Error df t value Pr(>\|t\|) | | | | |  |
| (Intercept) -2.045632 0.627069 418.317394 -3.262 0.001196 ** | | | | |  |
| log10BDNF 0.398496 0.203711 511.995382 1.956 0.050987. | | | | |  |
| Baseline age 0.020714 0.008251 161.147005 2.511 0.013041 * | | | | |  |
| Education 0.052976 0.022816 159.814033 2.322 0.021505 * | | | | |  |
| SexM -0.329554 0.127759 158.302211 -2.580 0.010804 * | | | | |  |
| CD4_CD8_ratio 0.299416 0.142597 556.787384 2.100 0.036201 * | | | | |  |
| logRNA 0.004131 0.059247 499.232953 0.070 0.944440 | | | | |  |
| log10BDNF:Week24 0.059385 0.075241 480.701786 0.789 0.430348 |  |  |  |  |  |
| log10BDNF:Week48 0.127754 0.075703 480.166927 1.688 0.092142 . | | | | |  |
| log10BDNF:Week96 0.303524 0.080371 485.152759 3.777 0.000179 *** | | | | |  |
| --- |  |  |  |  |  |

**ProBDNF**

| Fixed effects: |  |  |  |  |
| --- | --- | --- | --- | --- |
| Estimate Std. Error df t value Pr(>\|t\|) | | | | |
| (Intercept) -0.82881 0.45737 209.64511 -1.812 0.0714 . | | | | |
|  |  |  |  |  |
| log10ProBDNF -0.09832 0.05769 421.97318 -1.704 0.0891 . | | | | |
| Baseline_age 0.01749 0.00794 150.89245 2.203 0.0291 * | | | | |
| Edu_yrs 0.04915 0.02181 149.02869 2.254 0.0257 * | | | | |
| SexM -0.24968 0.12372 150.81087 -2.018 0.0454 * | | | | |
| CD4_CD8_ratio 0.31244 0.12791 483.23050 2.443 0.0149 * | | | | |
| logRNA -0.01311 0.04138 400.06815 -0.317 0.7515 | | | | |
| log10ProBDNF:Week24 0.01975 0.06172 377.69374 0.320 0.7491 | | | | |
| log10ProBDNF:Week48 0.07472 0.06289 377.24421 1.188 0.2356 | | | | |
| log10ProBDNF:Week96 0.27386 0.06254 386.81823 4.379 1.54e-05 *** | | | | |
| --- |  |  |  |  |
| Signif. codes: 0 ‘***’ 0.001 ‘**’ 0.01 ‘*’ 0.05 ‘.’ 0.1 ‘ ’ 1 |  |  |  |  |

**S2 Serum mBDNF/ProBDNF Levels and Cognitive Performance Post-ART initiation with Time since ART initiation set as a continuous variable**

| **Sensitivity analysis Model 1, Bdnf on Composite cognitive score** | | | | | |  |
| --- | --- | --- | --- | --- | --- | --- |
| Estimate Std. Error t value p_value | | | | |  |  |
| (Intercept) -1.404057631 0.5253349039 -2.6726905 7.739239e-03 | | | | | | |
| log10BDNF 0.077083989 0.1560455504 0.4939839 6.215072e-01 | | | | | | |
| Baseline_age 0.020042660 0.0073971822 2.7094993 6.940485e-03 | | | | | | |
| Edu_yrs 0.046676292 0.0204608740 2.2812462 2.290058e-02 | | | | | | |
| SexM -0.266950140 0.1146586116 -2.3282171 2.024828e-02 | | | | | | |
| CD4_CD8_ratio 0.262744336 0.1172546349 2.2408013 2.542190e-02 | | | | | | |
| logRNA 0.040779702 0.0277336826 1.4704034 1.420024e-01 | | | | | | |
| log10BDNF: Week_numeric 0.003124025 0.0004344594 7.1906039 2.027790e-12 |  |  |  |  |  |  |
|  |  |  |  |  |  |  |
| **Sensitivity analysis Model 2, ProBdnf on Composite cognitive score** | | | | | |  |
| print(fixed_effects) | |  |  |  |  |  |
| Estimate Std. Error t value p_value | | | | | | |
| (Intercept) -0.946246289 0.4311412436 -2.194748 2.858290e-02 | | | | | | |
| log10ProBDNF -0.120499549 0.0451400679 -2.669459 7.813168e-03 | | | | | | |
| Baseline_age 0.017603850 0.0077245737 2.278941 2.303819e-02 | | | | | | |
| Edu_yrs 0.047322723 0.0212194338 2.230160 2.612405e-02 | | | | | | |
| SexM -0.249677884 0.1203908145 -2.073895 3.853595e-02 | | | | | | |
| CD4_CD8_ratio 0.285393802 0.1172125721 2.434840 1.520306e-02 | | | | | | |
| logRNA 0.033462867 0.0271835366 1.230998 2.188297e-01 | | | | | | |
| log10ProBDNF: Week_numeric 0.003011767 0.0004632946 6.500759 1.742274e-10 |  |  |  |  |  |  |
